# From Metagenomes to Functional Expression of Resistance: *floR* Gene Diversity in Bacteria from Salmon Farms

**DOI:** 10.3390/antibiotics14020122

**Published:** 2025-01-24

**Authors:** Javiera Ortiz-Severín, Iñaki Hojas, Felipe Redin, Ervin Serón, Jorge Santana, Alejandro Maass, Verónica Cambiazo

**Affiliations:** 1 Laboratorio de Bioinformática y Expresión Génica, Instituto de Nutrición y Tecnología de los Alimentos, Universidad de Chile, Santiago 7830490, Chile; felipe.redin@ug.uchile.cl (F.R.); vcambiaz@inta.uchile.cl (V.C.); 2 Centro de Modelamiento Matemático, Universidad de Chile and UMI-CNRS 2807, Santiago 8370415, Chile; ihojas92@gmail.com (I.H.); amaass@dim.uchile.cl (A.M.); 3 Millennium Institute Center for Genome Regulation, Santiago 7850000, Chile; 4 Etecma EIRL, Puerto Montt 5500001, Chile; ervin.seron@etecma.cl (E.S.); jsantanavet@gmail.com (J.S.); 5 Departamento de Ingeniería Matemática, Facultad de Ciencias Físicas y Matemáticas, Universidad de Chile, Santiago 8370415, Chile

**Keywords:** salmon aquaculture, marine bacterial communities, metagenomics, florfenicol, antibiotic resistance, *floR* gene, gene variants

## Abstract

**Background.** The increase in antibiotic resistance in human-impacted environments, such as coastal waters with aquaculture activity, is related to the widespread use of antibiotics, even at sub-lethal concentrations. In Chile, the world’s second largest producer of salmon, aquaculture is considered the main source of antibiotics in coastal waters. In this work, we aimed to characterize the genetic and phenotypic profiles of antibiotic resistance in bacterial communities from salmon farms. **Methods.** Bacterial metagenomes from an intensive aquaculture zone in southern Chile were sequenced, and the composition, abundance and sequence of antibiotic resistance genes (ARGs) were analyzed using assembled and raw read data. Total DNA from bacterial communities was used as a template to recover *floR* gene variants, which were tested by heterologous expression and functional characterization of phenicol resistance. **Results.** Prediction of ARGs in salmon farm metagenomes using more permissive parameters yielded significantly more results than the default Resistance Gene Identifier (RGI) software. ARGs grouped into drug classes showed similar abundance profiles to global ocean bacteria. The *floR* gene was the most abundant phenicol-resistance gene with the lowest gene counts, showing a conserved sequence although with variations from the reference *floR*. These differences were recovered by RGI prediction and, in greater depth, by mapping reads to the *floR* sequence using SNP base-calling. These variants were analyzed by heterologous expression, revealing the co-existence of high- and low-resistance sequences in the environmental bacteria. **Conclusions.** This study highlights the importance of combining metagenomic and phenotypic approaches to study the genetic variability in and evolution of antibiotic-resistant bacteria associated with salmon farms.

## 1. Introduction

Aquaculture is an expanding industry, increasingly recognized for its key role in food security and nutrition and driven by the steady growth in production in countries such as Chile, China and Norway [1]. Aquaculture has grown dramatically in recent decades and has become an important component of global fish production to meet human consumption demands. In the year 2022, aquaculture production reached 94.4 million tons of aquatic animals, surpassing capture fisheries for the first time [2]. This intensification in aquaculture production has led to the emergence and re-emergence of infectious diseases and to a significant increase in the use of antibiotics [3]. Thus, aquaculture production systems have been identified as “hotspots” of antimicrobial resistance [4], where the widespread use of antibiotics increases the selective pressure on bacterial populations and also increases the rates of horizontal transfer between environmental bacteria and animal pathogens [3,5,6]. It is important to note that the continuous dissemination of antibiotics through water alters the ecological balance of the marine microbiome, with important environmental consequences including the loss of biodiversity and the emergence of antibiotic-resistant bacteria (ARB) [7,8,9]. The continuous use of antibiotics in production systems appears to be an important driver for the evolution, proliferation and spread of antibiotic resistance genes (ARGs) and may contribute significantly to the increased levels of antibiotic resistance in the environment [10].

Salmon farming was introduced in Chile in the late 1980s, and intensive aquaculture practices have made Chile the second-largest producer of salmon in the world [1,11]. As a result, susceptibility to a variety of infectious diseases has increased, and more than 400 tons of antibiotics are used each year to control bacterial diseases in salmon reared in marine aquaculture facilities. From 2015 to date, florfenicol (FFC) has been the drug of choice to treat bacterial outbreaks, accounting for 95.5% of antibiotics delivered by food during 2023 [12]. More than 90% of all antimicrobials used in Chilean aquaculture are used for treating Salmonid Rickettsial Septicemia (SRS) or piscirickettsiosis [12], a chronic bacterial disease caused by the Gram-negative bacterium *Piscirickettsia salmonis* with high prevalence in Chile [13]. SRS is the most serious infectious disease for the salmon farming industry in Chile, affecting fish during the seawater production cycle stage with high mortality (44.7% in year 2023 [14]). Therefore, the widespread use of FFC poses a threat to the development of antimicrobial resistance in bacteria. FFC is a fluorinated structural analog of thiamphenicol and chloramphenicol and is a broad-spectrum antibiotic that is the most widely used in aquaculture worldwide [15]. The problem of FFC resistance is becoming increasingly serious; previous work has shown that this antibiotic can persist in the seawater column at sub-lethal concentrations after six months following oral FFC treatment and, due to its low sorption tendency, can remain suspended under the influence of currents [16,17]. Therefore, remaining antibiotics in the environment promote the spread of florfenicol resistance genes and induce mutation-driven resistance [18]. The *floR* gene was the first florfenicol-resistance gene discovered and is currently the most widespread. An increasing number of drug-resistant bacteria have been found in the rearing environment and in animals [19,20,21], suggesting possible transmission between animals, humans and the surrounding environments once these genes spread, posing a major threat to public health. Antimicrobial resistance (AMR) has been recognized by the World Health Organization (WHO) as a major threat to global public health [22]. As a result of aquaculture activity, discharges of chemicals, antibiotics, excessive nutrients or microbial pathogens, among other factors, have the potential to adversely impact the physicochemical environment around farms. In this scenario, ARB and ARGs within aquaculture systems are of great concern largely due to the intensive use of antibiotics to treat disease [23,24].

In a previous work, we demonstrated an increased adaptation to antibiotics and a higher proportion of multi-resistant bacteria isolated from the surface seawater of a salmon farming area compared to a distant, undisturbed site [25]. In the same work, metagenomics data revealed a significant higher abundance of antibiotic resistance genes conferring resistance to 11 antibiotic families in the bacterial community from salmon farms, indicating that the overall proportion of bacteria carrying the resistance determinants was higher in salmon farms than in the undisturbed site.

In this work, we investigated the prevalence of florfenicol-related resistance genes in bacteria from two salmon farms located in a highly productive area of southern Chile [26]. Using metagenomic analysis together with phenotypic experimental approaches, we aimed to explore the antibiotic resistance landscape of the bacterial communities present in the salmon farms in direct contact with the farmed salmons and the impact of long-lasting FFC treatment on the ARG repertoire of these bacteria. We aimed to use the metagenomic data to search for specific ARG sequences to serve as a basis for specific experimental approaches and, thus, to find the relationship between gene prediction and the antibiotic resistance level (phenotype) in bacteria.

## 2. Results and Discussion

### 2.1. Bacterial Community Composition

**Figure 1:**
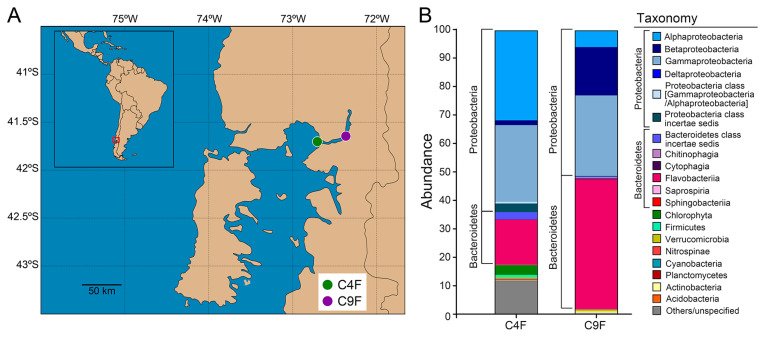
Characteristics of surface seawater bacterial samples from two fish farms in southern Chile. (**A**) Geographical location of surface seawater samples collected from salmon farms in autumn (C4F) and spring (C9F). (**B**) Taxonomy description (mOTUs) of samples C4F and C9F at phylum level and class level for the two most abundant phyla (Proteobacteria and Bacteroidetes).

The bacterial fraction was collected from the surface seawater of two fish farms located in the northern Patagonian region of Chile (Figure 1A), one in a sheltered marine bay during the autumn season (C4F) and one in an estuarine site during the spring season (C9F). The sites differed mainly in salinity and the fish species, although the physicochemical parameters of the water in both sites were adequate for the normal development of the fattening phase (Table 1). In addition, both fish farms reported the use of oral florfenicol to treat bacterial diseases in the three months prior to sampling, but only C4F had an outbreak of SRS at the time of sampling (Table 1).

Following metagenomic sequencing, the mOTUs2 marker gene cluster-based approach was used for taxonomic profiling of the bacterial communities. The normalized relative abundances of the C4F and C9F bacterial communities were compared, as shown in Figure 1B. The surface seawater bacterial communities were dominated by the phyla Proteobacteria (64.06% in C4F and 51.48% in C9F) and Bacteroidetes (18.73% in C4F and 46.92% in C9F), in particular, the classes Alphaproteobacteria, Betaproteobacteria, Gammaproteobacteria (31.6%, 1.70% and 27.09% in C4F and 5.74%, 17.07% and 28.4% in C9F, respectively) and Flavobacteriia (15.95% in C4F and 45.91% in C9F, Figure 1B). The predominance of Alpha- and Gammaproteobacteria (Proteobacteria) and Flavobacteriia (Bacteroidetes) in coastal and estuarine environments with active aquaculture has been reported previously [25,27]. Interestingly, *Piscirickettsia salmonis* was one of the mOTUs identified in C4F, which correlated with an SRS outbreak in this salmon farm, but it was not identified in C9F (Table 1).

### 2.2. Prediction of ARGs in Metagenomes

**Figure 2:**
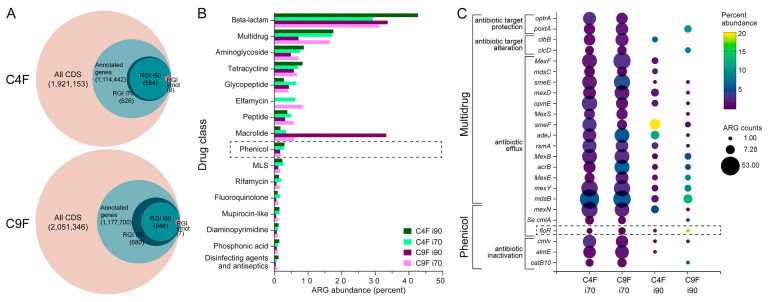
ARG prediction in metagenomes. (**A**) Metagenomic results. Concentric circle scheme of gene annotation results and ARG prediction using RGI tool with different parameters (RGI default cutoff value “perfect” or exploratory settings RGI with 90% identity threshold (i90) and RGI with 70% identity threshold (i70)). (**B**) Classification of predicted ARGs into drug classes. Percentages are shown in the bar graph for drug classes above 1% abundance. (**C**) Abundance of genes conferring resistance to phenicol antibiotics. Bubble plot of the ten most-represented ARGs conferring resistance to phenicols. Gene abundance (as a percentage of the total phenicol drug class) is shown in color, and the number of genes in each metagenome is represented by the size of the circle.

Metagenomic sequencing and annotation yielded a total of 1,921,153 coding DNA sequences (CDS) in C4F and 2,05,346 in C9F (Figure 2A). Antibiotic resistance genes (ARGs) were predicted using the Resistance Gene Identifier (RGI) software with standard parameters (cutoff value set to “perfect” [28]) or in the exploratory mode by filtering “strict” and “loose” results by 90% identity (i90) and 70% identity (i70). As shown in Figure 2A, lowering the threshold to i70 increased the number of ARGs in both metagenomes by 7.2% and 23.7% for C4F and C9F, respectively. Interestingly, the results obtained with the default RGI parameters were less than 0.001% of the annotated genes for both samples, whereas the more permissive i90 and i70 thresholds increased the number of ARGs to more than 0.05% of the annotated genes in C4F and between 0.046% and 0.058% in C9F, highlighting the importance of optimizing search parameters for environmental samples [29]. Differences in i70 and i90 results can also be seen in the ARGs classified by drug class (Figure 2B). The multidrug classification increased in both samples by using the i70 threshold, and additional classifications appeared, such as rifamycin, salicylic acid and isoniazid-like antibiotics (Table S1). The most notable dissimilarity between the i90 and i70 results corresponded to macrolides in C9F, whose abundance increased from 1.86% to over 33.2% when using the i90 threshold. This was consistent with an increase in the number and abundance of ARGs in the multidrug class when using the i70 threshold. However, the overall trend of the most abundant drug classes remained similar for i90 and i70, with beta-lactam, multidrug, aminoglycoside, tetracycline and glycopeptide antibiotics accounting for over 65% of the ARG abundance (except for C9F ARGs with the i90 threshold). Similar to our observations, previous work by Xu et al. [30] examined metagenomic and metatranscriptomic data from different ocean depths across all major oceanic regions and reported that the most abundant drug classes in seawater bacteria were beta-lactams, multidrug, aminoglycoside and tetracyclines [31]. Consistent with our result, Xu et al. found that the phenicol drug class was the ninth most represented in seawater metagenomes [31]. Considering the diversity of samples analyzed by Xu et al. and the fact that our samples were taken from anthropogenically and antibiotic-impacted environments (fish farms), the distribution of the abundance of ARGs sorted by drug class seems to be a characteristic of seawater bacterial communities regardless of environmental conditions. Alternatively, the composition of the database is biased towards clinically relevant pathogens, which may hinder the gene prediction for environmental bacteria [29]. However, given the extent of florfenicol use at the sampling sites and previous reports of increased abundance of phenicol ARGs in seawater bacteria from salmon farms [25], we explored the specific ARG composition of the phenicol drug class in C4F and C9F to assess the relative abundance of *floR*, the most prevalent phenicol resistance gene in fish farms [18,32,33].

Phenicol resistance genes were classified into two subgroups: genes conferring resistance to phenicols only and multidrug resistance genes (Figure 2C). As observed for all drug classes (Figure 1B), multidrug ARGs conferring resistance to phenicols increased in number and abundance when the i70 threshold was used. Multidrug ARGs represented the most abundant and most repeated (in gene counts) phenicol resistance genes in both samples, and their main resistance mechanism was antibiotic efflux, followed by antibiotic target protection and target alteration. Conversely, phenicol-specific genes were less represented, but, interestingly, the most abundant ARG when using more stringent parameters (i90) was *floR*, although the number of gene counts was similar between i70 and i90 (two vs. one for C4F and four vs. one for C9F, respectively). This was particularly the case for *floR*, as all other ARGs evaluated had substantially higher counts when using the i70 threshold (from 5 to 53, Figure 2C). Thus, the *floR* sequence appeared to be conserved, present in low numbers (one to four counts) and in high gene abundance.

#### Sequence Analysis of *floR* Gene in Bacterial Metagenomes

**Figure 3:**
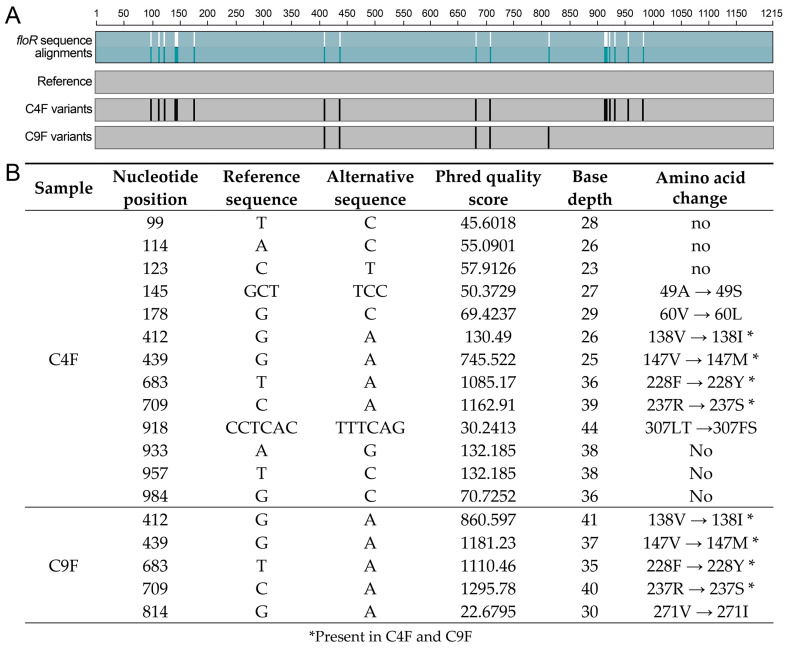
Nucleotide variations in metagenomic sequences of the *floR* gene. (**A**) schematic representation of nucleotide alignment of the *floR* reference gene and the nucleotide changes (variants) found in the C4F and C9F metagenomes. (**B**) SNP base-calling results for C4F and C9F metagenomes. The table shows specific nucleotide changes in metagenomic sequences for each sample (C4F and C9F), the nucleotide position in the gene, the reference nucleotide identity for each position, the alternative sequence (nucleotide change found in each metagenome), the phred quality score and base depth for each alternative nucleotide and the corresponding amino acid change.

Closer examination of *floR* revealed discrepancies between the Comprehensive Antibiotic Resistance Database (CARD) reference sequence (*Escherichia coli* AF231986.2 *floR*) and C4F and C9F *floR* (complete sequences in Table S2). In C4F, three base changes were identified at position 439 (G → A), position 683 (T → A) and position 709 (C → A). In addition, in C9F, a fourth base change was identified at position 412 (G → A). A BLASTN search performed against the GenBank database showed that the C4F *floR* sequence was identical to *floR* found in resistance plasmids from *E. coli* (plasmids pZ1323CEC0004-4 and pZ1323CEC0002-3), *Klebsiella pneumoniae* (plasmid pRWS291.s4), *Citrobacter tructae* (plasmid unnamed1), *Salmonella enterica* subsp. *enterica* serovar Typhimurium (plasmids pZ1323SSL0036-3, pZ1322SSL0073-2 and pZ1323SSL0039-3) and *Raoultella terrigena* (plasmid small unnamed1). The 412A nucleotide change from C9F has not been previously described. Given the bias that is introduced by the assembly process in the construction of longer contigs, we further investigated these nucleotide changes in the *floR* gene sequences by detecting genetic variants as single-nucleotide polymorphisms (SNPs). For this purpose, the raw reads from the C4F and C9F metagenomes were mapped to the reference *floR* sequence from *E. coli* AF231986.2 to reconstruct the *floR* gene, considering both the identity and the coverage of each nucleotide in the 1215 bp gene. All possible nucleotide changes between the reference sequence and the *floR* reconstructed from metagenomes were subjected to a statistical test to estimate the probability that a base was incorrectly called (see Section 3). Thus, we constructed a chimeric sequence of *floR* for each metagenome containing all the nucleotide changes identified by the read’s information (Figure 3A).

As shown in Figure 3, thirteen nucleotide positions with changes between the metagenome and the reference sequence of *floR* were identified for C4F with a phred score greater than 20 and base depth greater than 23 (highly representative changes). Of these, only seven nucleotide changes were translated into amino acid changes in the final protein (Figure 3B). These included the above changes identified by RGI i70, with the addition of four other variants (including the 412A nucleotide change previously identified in C9F) and six silent changes. Five positions with nucleotide differences were identified in the C9F metagenome, all of which were translated into amino acid changes, and four of them were present in C4F as well. Four of these changes corresponded to the RGI i70 changes, with the addition of the 814A variant. Thus, by further inspecting nucleotide sequences using the raw reads from the metagenomes, SNP base-calling was successful in uncovering novel gene variability in the metagenomic samples when compared to the RGI tool, which predicted only one *floR* sequence per sample. SNP base-calling also allowed for identifying underrepresented variants (such as 412A in C4F), silent mutations in C4F and new positions with nucleotide variants (such as 145TCC, 178C, 984C and 918TTTCAG).

All the variants listed in Figure 3B were aligned to the assembled metagenomes in a search of contigs containing *floR* variants (Table 1). As a result, one to three contigs containing the *floR* sequence were identified in C9F (NODE_34943) and C4F (NODE_20705, NODE_566496 and NODE_1421360). Prior ARG prediction with RGI i70 found four contigs with *floR* in C9F and two in C4F. The best-scoring variants (Figure 3B) mapped against the assembled contigs found an exact match to the best-hit contig identified by RGI (99–100% sequence identity, Table 1), thus validating the accuracy of the variant or SNP base calling. Common approaches to the detection of SNPs and indels in metagenomic data use the analysis of un-assembled reads or gene sequences in assembled contigs [34,35]. The use of reads for gene sequence determination allows for the identification of sequence variants but does not provide information about the genetic context. The RGI tool works on a contig-based gene search, and although assembly produces larger genomic fragments, it increases the risk of producing chimeric contigs with a single gene variant representing the most probable sequence (consensus k-mer) [34], thus misrepresenting the original sample and losing variability information. On the other hand, the predominant model for working with short reads is to have one assembled genome against which all others are compared. Here, we took advantage of conventional tools to identify sequence variants using metagenomic reads mapped against a reference gene. We therefore propose a base-calling method for finding gene variants in assembled metagenomes by using complementary approaches that combine gene variant analysis obtained from reads with assembled contig information to identify the genetic context of the variants.

**Figure 4:**
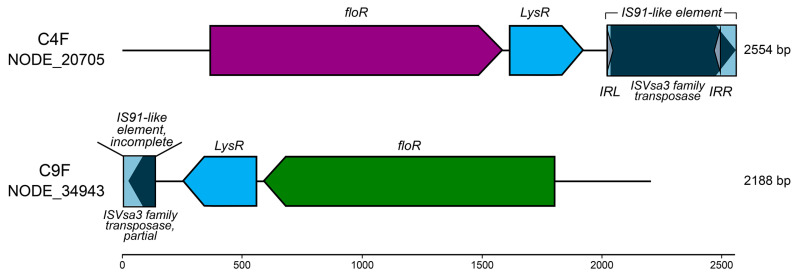
Schematic genetic context of *floR* in assembled contigs. Comparison of contigs containing *floR* genes in marine bacteria from the aquaculture sites. The best contigs for each metagenome were selected based on gene identity and completeness. The arrow heads indicate the direction of transcription/translation. The putative insertion sequences (IS91-like elements) are shown as boxes, and the transposases (IS*Vsa3* family transposases, dark blue) and the short terminal inverted repeats (IRL and IRR, grey arrowheads) are shown inside the IS boxes.

We further examined the gene content of the assembled contigs containing complete *floR* sequences (100% coverage in Table 2). Only one contig for C4F and C9F had more than one gene sequence, and they had the same gene content and configuration (NODE_20705 in C4F and NODE_34943 in C9F, Figure 4). The genetic context included the *floR* gene in the 5′ end, followed by a *LysR* regulator and a putative transposase sequence (incomplete in C9F) with homology to IS*Vsa3* family transposases belonging to IS91-like elements (Figure 4). Interestingly, all genes were present in the same open reading frame, and the intergenic space was conserved between both contigs (28 bp between *floR* and *LysR* and 112 bp between *LysR* and the transposase gene). The IS91 family insertion sequences (ISs) are characterized by carrying an open reading frame (ORF) encoding an HUH Y2 transposase [36]. Passenger genes such as transcription regulators, methyltransferases and antibiotic resistance can be located upstream, downstream or on both sides of the transposase gene [36,37]. Here, we observed the presence of an IS91-like family element located downstream of the *floR* resistance gene and the *LysR* regulator (Figure 4). The putative IS contained a complete IS*Vsa3* transposase and two flanking inverted repeats sequences in the C4F metagenome and an incomplete IS*Vsa3* transposase without flanking repeats in C9F (Figure 4). Even though more analyses are needed to confirm the location of *floR* in mobile elements or if *floR* is a passenger gene of the IS, the presence of a transposase (and inverted repeats in the C4F contig) suggests that this could be a mobilizable sequence or that mobilization of *floR* has occurred.

The new variants found in C4F and C9F did not match any known *floR* sequence with 100% identity. As discussed in previous works, due to the sequence similarity of ARGs between multiple genomic contexts across different species, the assembly of contigs in these ARG-containing regions is difficult and, therefore, the accuracy of capturing the genomic context (and by extension, the taxonomic profiling) is very low [38]. Consequently, the *floR*-containing contigs found in this work were relatively short, less than 3 kb. Therefore, we performed BLAST searches of individual genes against the GenBank database and found that both the IS91 family transposase and the *LysR-like* gene were identical to Gammaproteobacteria genes from known pathogens such as *E. coli*, *K. pneumoniae*, *S. enterica*, *Acinetobacter baumanii* and *Providencia rettgeri*, among others. This, together with the conserved gene sequence and genetic map of the *floR*-containing contigs, and the fact that all variants mapped to the same contig in each sample, suggests that *floR* belongs to closely related bacteria of the Gammaproteobacteria class at both sites (C4F and C9F).

Early reports of florfenicol resistance in bacteria described the *floR* gene in plasmids or other mobile elements, such as integrons and transposons [20,21,39,40,41,42]. The IS91 transposon [43], a highly conserved Tn4371-like ICE [42], and the ISCR2 insertion sequence harboring the Tn21 transposon [44] have been reported to mobilize *floR* in bacteria. It is thought that the antibiotic resistance passenger genes in these ISs are transmitted during the transposition. These IS*Vsa3*-containing ISCRs have the potential to transfer multiple antibiotic resistance genes into a variety of bacteria and integrate into plasmids and chromosomes [37,45]. In *E. coli* BN10660, the plasmid-borne *floR* gene is part of a functional transposable element containing a *tnpA* transposase and a putative *LysR-like* transcriptional regulator [46], and the same configuration has been described in *Pasteurellaceae* plasmids [47]. This configuration is consistent with our results for *floR* in the bacterial metagenomes from salmon farms (Figure 4). The LysR-type transcriptional regulator (LTTR) family of proteins is a well-known group of transcriptional regulators involved in diverse physiological functions. Previous work found that these LTTR family proteins were downregulated following antibiotic treatment in *Aeromonas hydrophila*, a Gram-negative multi-resistant bacterium that causes serious infections in aquaculture, suggesting that they may be involved in the regulation of antibiotic resistance [48]. LysR-type transcriptional regulators associated with five major facilitator superfamily (MFS) multidrug transporters were identified in *Vibrio cholerae* [49]. All the MFS genes were regulated by their corresponding LysR-type MFS regulator, and deletion of these regulators resulted in increased susceptibility to antibiotics [49]. Accordingly, the gene configuration of *floR*-containing contigs in the salmon farm bacterial metagenomes suggests that *floR* expression is regulated by the putative LysR-like transcriptional regulator and mobilizable by the contiguous transposase, at least in C4F, as the transposase sequence was incomplete in the C9F contig.

The *floR* abundance information, together with the conserved gene sequence and the similarity of *floR*-containing contigs in the surveyed metagenomes, suggest that the *floR* gene is carried by closely related bacteria that are present in relatively high abundance in seawater communities. However, there is some gene variability, as corroborated by the sequence variants found using the raw-read information. Although this approach allowed us to find more *floR* gene variants, it is not possible to know whether the gene sequences in the bacterial cells contained all the predicted nucleotide changes or a combination of them. Therefore, we sought to amplify and clone the *floR* gene present in the C4F and C9F bacterial communities to study both their sequence and antibiotic resistance profiles.

### 2.3. Identification and Analysis of floR Variants

#### 2.3.1. Cloning and Sequence Analysis of *floR* Variants

To further verify the *floR* gene sequence, PCR primers, based on the *E. coli* AF231986.2 *floR* reference sequence, were used to amplify a 1.2 kb PCR product from total DNA purified from C4F and C9F seawater bacteria. The complete ORFs of the *floR* sequences were cloned in a pETDuet-1 vector and transformed into *E. coli* BL21 (DE3). In addition, the AF231986.2 *floR* reference sequence was cloned as a control (Figure S1) for minimum inhibitory concentration (MIC) experiments.

**Figure 5:**
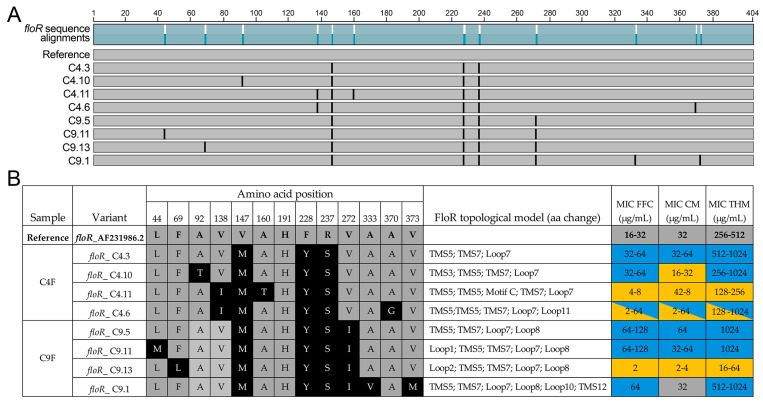
Phenotypic characteristics of the FloR variants. (**A**) Schematic representation of the protein alignment of the FloR reference protein sequence and the sequence variants found in the C4F and C9F clones. (**B**) Table showing the FloR variants identified in the clones from each sample (C4F and C9F), the amino acid position of the aminoacidic change in the translated protein, the location of the aminoacidic change in the FloR topological model, according to Braibant et al.’s protein model [51], and the MIC results for the eight clones with *floR* gene variants from C4F and C9F to three phenicol antibiotics (FFC, florfenicol; CM, chloramphenicol; THM, thiamphenicol). The reference amino acids are shown in gray, and the changes in the variants are highlighted in black. In the MIC results, gray represents the reference values (MIC for FloR reference sequence), blue represents an increase in MIC values and yellow represents a decrease.

A total of fourteen clones were sequenced, and eight clones with different *floR* sequences were found in both samples (four for C4F and four for C9F, Table S2). None of the clones in the present study showed 100% sequence identity to the reference *floR* sequence, exhibiting from three to five mismatches in the nucleotide sequence, corresponding to three to five amino acid sequences (Figure 5A). A BLASTN search against the GenBank database revealed that eight of the nine variants were novel *floR* sequences, and only variant *floR*_C4.3 had 100% sequence identity to other known bacterial species. This variant was identical to the *floR* C4F sequence found by RGI and was thus identical to *floR* in plasmids from enterobacterial species such as *E. coli* (CP148486.1, CP148500.1), *S. enterica* subsp. *enterica* serovar Typhimurium (CP149322.1, CP149319.1, CP149292.1), *R. terrigena* (CP145802.1) and *C. tructae* (CP038468.1). Interestingly, *C. tructae* is a novel *Citrobacter* pathogenic species isolated from the kidney of diseased rainbow trout (*Oncorhynchus mykiss*) from a trout farm in Korea [50]. The other seven clones (variants *floR*_C4.6, *floR*_C4.10, *floR*_C4.11, *floR*_C9.1, *floR*_C9.5, *floR*_C9.11 and *floR*_C9.13) had no exact match in the databases. Remarkably, nucleotide changes 439A, 683A and 709A were present in all sequenced clones (translated as 147M, 228Y and 237S, respectively, Figure 5B). These changes were also observed in the metagenomic information (Figure 3B). In addition, 412A (138I), predicted by metagenomic sequencing for C4F and C9F (Figure 3B), was found only in C4F clones (variants *floR*_C4.6 and *floR*_C4.11, Figure 5B), whereas a nucleotide change in 814A (272I), also predicted by metagenome sequencing, was consistently identified in all C9F clones, thus suggesting a conserved variant in the C9F bacterial community. The other nucleotide variants shown in Figure 3B were not identified in the C4F and C9F clones, although more cloning and sequencing effort could be carried out to search for underrepresented variants. Thus, as expected, no clones were found that carried only one nucleotide change in the *floR* sequence, but rather a combination of changes that were conserved (147M, 228Y and 237S for both sites and 272I for C9F) or unique variants (138I and 370G in *floR*_C4.6, 92T in *floR*_C4.10, 138I and 160T in *floR*_C4.11, 333V and 373M in *floR*_C9.1, 44M in *floR*_C9.11 and 69L in *floR*_C9.13) were found.

#### 2.3.2. Characterization of FloR Variants

The *floR*_C4.3 and *floR*_C9.5 clones carried the representative C4F and C9F FloR sequences, respectively. According to the topological model of FloR by Braibant et al. [51], FloR belongs to the MF superfamily of efflux transporters and is formed by twelve trans-membrane sequences (TMS) and eleven loops, with five conserved motifs. According to our results, the C4F and C9F clones presented amino acid changes in TMS5, TMS7 and loop7 in addition to loop 8 in the C9F clones (Figure 5B). To assess whether these changes were responsible for alterations in antibiotic resistance profiles, we determined the MICs of the clones and control strains against a group of phenicol drugs (Figure 5B and Figure S1). Apart from *floR*_C9.13, all *floR*-bearing clones showed increased MIC values for the three antibiotics tested (florfenicol, FFC; chloramphenicol, CM; thiamphenicol, THM) compared to the control strains (*E. coli* ATCC 25922 as an MIC assay internal control, *E. coli* BL21 (DE3) wild-type and *E. coli* BL21 (DE3) carrying an empty ETDuet-1 vector). The reference FloR sequence showed MIC values similar to those expected for FFC, CM and THM [51]. The representative C4F and C9F FloR sequences (clones *floR*_C4.3 and *floR*_C9.5) had increased MIC values by 1–2-fold compared to the reference FloR (Figure 5B) in all three phenicol antibiotics. Thus, 147M, 228Y and/or 237S modifications were responsible for this increased antibiotic resistance, although it is not known if all contributed equally or only one modification was responsible for this increased resistance. In addition, 272I, the conserved change in all C9F clones (and metagenomic *floR* sequence) had no apparent effect on resistance, with the exception of FFC MIC, which was 1-fold higher than in the *floR*_C4.3 clone. However, additional sequence modifications reduced this high-antibiotic resistance phenotype. For example, substitution of non-polar amino acids with hydrophobic side chains 333A → V in loop 10 and 373V → M in TMS12 (COOH terminal part of the protein) only decreased the MIC of CM to that of the reference protein. However, substitution of a non-polar amino acid with a hydrophobic side chain by a polar uncharged side chain amino acid such as the 92A → T modification in TMS3 (*floR*_C4.10 clone) further decreased the MIC of CM to half the reference value. More drastic was the effect of modifying a phenylalanine for a leucin in loop 2 (69F → L in the *floR*_C9.13 clone) and the addition of a polar uncharged side chain in motif C (160A → T in the *floR*_C4.11 clone), which reduced the MIC values for all three antibiotics to levels similar to the wild-type strain (empty vector or no vector, Figure S1). The effect of the 138V → I modification present in the *floR*_C4.6 and *floR*_C4.11 clones was not apparent. However, the addition of the 370G change in the *floR*_C4.6 clone caused variability in the MIC results, as different replicates showed different MIC values (indicated by yellow and blue colors in the MIC results, Figure 5B). A total of 14 independent replicates were evaluated for the *floR*_C4.6 clone, and about half of them had higher MIC values than the reference FloR (32–64 µg/mL for FFC, 32–64 µg/mL for CM and 256–1024 µg/mL for THM), while the other half were closer to wild-type strains without FloR (2–4 µg/mL for FFC, 2–4 µg/mL for CM and 128 µg/mL for THM). Previously, a FloR-68D-phoA fusion protein was studied [51], and this position was found to correspond to a periplasmic residue, which would also be true for 370G in the *floR*_C4.6 clone. This work did not report the effect of amino acid modification in the periplasmic location, so to our knowledge, this is the first report of a mixed phenotype (susceptible/resistant phenotype) caused by the substitution of a non-polar amino acid with a hydrophobic side chain for a polar residue (370A → G in *floR*_C4.6 clone) in the periplasmic moiety of FloR. However, further experiments and analyses are needed to determine the exact effect of these amino acid substitutions on protein structure and function.

In biological scenarios, random genome mutations and genetic drift underlie the evolution of antibiotic resistance. Evolutionary experiments in *Pseudomonas aeruginosa* under antibiotic pressure showed that drug resistance is influenced not only by the presence of the antibiotic but also by the drug concentration, population genetics, size and events such as bottlenecks that occur during an infection of a host, by physical changes in the environment and as a consequence of antibiotic treatment [52]. Mahrt et al. found that antibiotic resistance in bacteria is favored as a result of high antibiotic selection but also under low antibiotic selection and severe bottlenecks. In addition, this condition favors the increase in and spread of low-resistance variants that exhibit highly competitive fitness in the absence of high antibiotic pressure [52]. Furthermore, rifampicin resistance studies in *P. aeruginosa* showed that mutations that confer low levels of antibiotic resistance do not restrict the ability to evolve high levels of resistance, since in resistance evolution, many beneficial mutations are available, and genotypes with low levels of resistance rapidly acquire secondary resistance mutations that confer very high levels of antibiotic resistance [53]. In this work, the two salmon farms studied were subjected to oral FFC treatments within the last three months and therefore, as mentioned above, sub-lethal concentrations of FFC in the water column [16]. Experimental approaches to study the response of bacterial communities to florfenicol treatment in controlled scenarios, such as aquatic microcosms [54] and tank production systems [18], have shown that the addition of florfenicol led to increased abundance of the *floR* genes by selection and increased rates of horizontal transfer, but it also induced mutation-driven resistance, as observed by increased frequencies of nucleotide polymorphisms. Here, we observed a mixture of *floR* sequences with a correspondent mixture of antibiotic resistance profiles, showing conserved nucleotide and amino acid variations (such as 147V → M, 228F → Y and 237R → S for both sites and 272V → I for C9F) and unique variations in different clones, which not only led to increases in antibiotic resistance for three phenicol antibiotics (FFC, CM and THM) but also increased susceptibility to one or more of them. This coexistence of both resistant and susceptible variants in closely related bacteria in the same community could be the effect of low antibiotic pressure allowing for the occurrence of nucleotide changes in gene sequences by the accumulation of point mutations and the spread of variants with low-level antibiotic resistance but higher competitive fitness during weak bottlenecks and weak selection [52], situations likely to be encountered by seawater bacteria after antibiotic treatments. The understanding that shared environments and physiological similarities between species facilitate the spread of pathogens and promote the rise of antibiotic-resistant bacteria has created the need for an integrated approach aimed at sustainable practices in aquaculture [23,55]. Thus, the use of antibiotics and their spread into the surrounding environment should be avoided in the future design of aquaculture systems based on the One Health initiative.

## 3. Materials and Methods

### 3.1. Experimental Design and Sampling

Two sampling sites in southern Chile were chosen in an area of intensive aquaculture in the Los Lagos region. The geographical location of the salmon farms is depicted in Figure 1A; the map was created using python with standard cartopy tiles and GPS metadata.

The surface seawater from the two salmon farms (C4F and C9F) was collected in May and October 2022 (autumn and springtime) in areas that were approximately 30 m deep using sterile opaque plastic containers. At both sites, about 20 L of surface seawater was collected in different sampling points from each site to create replicates. The water physicochemical parameters were obtained with a HI-98194 multiparameter device (Hanna Instruments, Woonsocket, RI, USA). Measurements were taken concurrently with the water sample collection and are shown in Figure 1B. The pico-nanoplanktonic fraction (cell sizes between 0.22 nm and 3 µm) were obtained using a sterile filtration system composed of three filters with different pore sizes (200 µm nylon mesh, 3.0 μm sterile Whatman filter and 0.22 μm Sterivex filter). Five sterivex filters were obtained per site (4 L each) and were preserved in RNA Save (Biological Industries, Kibbutz Beit Haemek, Israel) for transportation and storage at −80 °C until processing.

### 3.2. DNA Purification and Sequencing

Sterivex filters were thawed on ice and gently washed with sterile PBS to remove the RNA Save solution. Filters were processed according to Cruaud et al. [56] by opening the sterivex cartridge and cutting it into slices before being introduced into sterile 1.5 mL tubes. DNA was purified using Fast DNA Stool kit (Qiagen, Hilden, Germany) with some modifications. Sterivex slices were suspended in ATL buffer (Qiagen), and 1 mg/mL lysozyme was added before incubation at 37 °C for 1 h. Proteinase K (2 mg/mL) was added to the mixture and incubated for 20 min at 56 °C, followed by a 5 min incubation with RNase (0.1 mg/mL, Omega-Biotek, Norcross, GA, USA) at room temperature. The mixture was homogenized thoroughly before following the column steps in the Fast DNA Stool kit protocol.

The DNA quality was evaluated by spectrophotometry (A260/A280 ratio) in an Infinite 200 PRO NanoQuant spectrophotometer (Tecan, Männedorf, Switzerland), and the integrity was verified by standard 1% agarose gel electrophoresis. DNA was suspended in ATE buffer (Qiagen) and quantified using a Qubit 2.0 Fluorometer (Thermo Fisher Scientific, Waltham, MA, USA). The purified DNA from marine bacterial communities from the C4F and C9F salmon farms was pooled and used for metagenomics library construction. Novogene (Sacramento, CA, USA) performed shotgun sequencing on the Illumina NovaSeq 6000 platform with a paired-end 150 bp configuration to obtain 12 Gb of raw data per sample. For the library construction, total DNA was randomly sheared, and then the smaller DNA fragments were end-repaired, A-tailed and further ligated with an Illumina adapter. The fragments with adapters were PCR-amplified, size-selected and purified. The library was checked and quantified with Qubit and real-time PCR, and a bioanalyzer was used for size distribution detection. Quantified libraries were pooled and sequenced.

### 3.3. Metagenome Assembly and Analysis

The quality of the raw metagenome sequences was assessed using the FastQC program v12.1 [57]. After identifying low-quality reads, the BBDuk tool v39.01 [58] was used to filter and trim the reads based on several parameters (minlen = 30 qin = 33 qtrim = lr trimq = 17\ktrim = r k = 17 mink = 13 hdist = 1). The reads were assembled into scaffolded contigs using meta-SPADES [59] with defined parameters (range of k-mers 21, 29, 39, 59, 79, 99), and resulting contigs of less than 550 bp were eliminated. The quality of the assemblies was evaluated using metaQUAST [60] with N50 metric scores above 1000, and coding sequences (CDS) were predicted using Prodigal v2.6.3 in “meta” mode. Functional annotation of bacterial metagenomes was performed on the contigs using eggNOG-mapper v2.1.12 (database v5.0.2) with default parameters [61]. IS elements were identified in the assembled contigs using ISEScan with default parameters [62].

### 3.4. Taxonomy Assignment in Metagenomes

Taxonomic profiling of bacterial metagenomes was based on metagenomic operational taxonomic units (mOTUs). High-quality reads in each sample were mapped to the mOTUs reference database using mOTUs profiler v3.1.0 with default parameters [63]. Relative abundance was standardized between 0 and 1 (using the scikit-learn MinMaxScaler algorithm in python) for each metagenome.

All mOTUs taxonomy results are listed in Table S3.

### 3.5. ARG Prediction, Relative Quantification and Abundance

Detection of ARGs in metagenomes was performed using RGI v6.0.1 (Resistance Gene Identifier) together with CARD v3.2.7 [28] using the assembled contigs. RGI was used with the default parameters, but additionally unfiltered results were obtained for the selection of ARGs with “perfect”, “strict” and “loose” cutoff values. For an exploratory search of ARGs, “strict” and “loose” were subsequently filtered by 90% (i90) and 70% (i70) sequence identity. All ARGs were further classified according to their resistance mechanism, family and drug class based on the CARD ARO terms. ARGs belonging to two or more drug classes were classified as multidrug.

To estimate the relative abundance of individual ARGs and ARGs classified into drug classes, reads were mapped to ARG-containing contigs using samtools [64,65] and BWA-MEM v0.7.17 [66], generating a deep coverage for each contig. Therefore, the estimated abundance was calculated as the mean contig coverage multiplied by the raw count of each target gene.

### 3.6. Analysis of floR Sequence Variants in Metagenomes

To identify gene variants in the metagenomic data, reads were mapped against the reference *floR* gene (*E. coli* AF231986.2) using BWA-MEM v0.7.17 [64,65]. Gene variants (SNPs and indels) were identified using FreeBayes v1.3.2 [67], filtering by high-quality scores (qual > 20) based on the nucleotide coverage evaluated as the phred score [68] of mapped reads, on nucleotides from variant sequences.

To correlate sequence variants with their genetic context, each *floR* variant (corresponding to the reference sequence containing one SNP) was mapped to previously annotated contigs to find the gene position in the contigs using BEDtools v2.30.0 [69].

### 3.7. PCR Amplification, Isolation and Sequencing of floR Genes

PCR reactions were carried out using Phire Hot Start II PCR Master Mix (Thermo Fisher Scientific) according to the manufacturer’s instructions. Oligonucleotides were synthesized by Macrogen (Seoul, Republic of Korea). The PCR-derived *floR* gene was obtained using floR_ATG_Fw and floR_stop_Rv primers listed in Table S4 in a Biorad thermocycler with the following cycling conditions: 1 cycle of 30 s at 98 °C; then, 35 cycles of 5 s at 98 °C, 5 s at 70 °C and 20 s at 72 °C; and finally, 1 cycle of 1 min at 72 °C. Amplification yielded a 1215 bp amplicon containing the entire length of the published gene sequence according to CARD (Genbank accession no. *E. coli* AF231986.2, 3308-4522). The template for this amplification was the total DNA of bacterial communities from the salmon farms (C4F and C9F). In addition, PCR primers were created to linearize the pETDuet-1 vector at the MCS1 sequence (pETDuet-1_Inverse_left and pETDuet-1_Inverse_right, Table S4). A Biorad thermocycler was used for the amplification of the 5420 bp PCR product with the following cycling conditions: 1 cycle of 30 s at 98 °C; then, 35 cycles of 5 s at 98 °C, 5 s at 60 °C and 80 s at 72 °C; and finally, 1 cycle of 1 min at 72 °C. Forty microliters of each PCR product were separated by 1% agarose gel electrophoresis at 90 V for 100 min. The appropriate bands were excised and purified utilizing a GeneJET Gel Extraction Kit (Thermo Fisher Scientific).

The purified PCR amplicons were used for subsequent PCR amplification to create fragments with overlapping ends for ligation with In-Fusion seamless cloning (Takara, Shiga, Japan). For that purpose, *floR* was amplified from the purified *floR* gene PCR product obtained from the total DNA of bacterial communities from the salmon farms using the frg_pET_floR_ATG_fw and frg_floR_stop_pET_rv primers (Table S4) with the same PCR conditions used for the first *floR* amplification, obtaining a 1255 bp amplicon. Ligation of *floR* sequences with overhangs to the linearized pETDuet-1 vector was conducted with In-Fusion seamless cloning (Takara) and transformed into *E. coli* HST08 Stellar chemically competent cells according to the manufacturer’s instructions. For each DNA template (C4F or C9F), 16 clones were selected, and their plasmids were purified to verify insert orientation. Routine PCR was carried out using GoTaq Green Master Mix (Promega, Madison, WI, USA) with the primers floR_med_Fw and AmpR_R1 to check for insert orientation (2355 bp amplicon; cycling conditions: 1 cycle of 2 min at 95 °C; then, 35 cycles of 30 s at 95 °C, 30 s at 58 °C and 2 min 22 s at 72 °C; and finally, 1 cycle of 5 min at 72 °C); to check for the correct *floR* gene length, primers floR_ATG_Fw and floR_stop_Rv (Table S4) were used with the same PCR conditions but using a 60 °C annealing temperature and 1 min 15 s at 72 °C for extension. Fourteen positive clones were sequenced by Sanger technology (Macrogen, Seoul, Republic of Korea) using universal primers pET-Upstream and DuetDOWN1 (Table S4). Sequence alignments, comparisons and analysis were carried out using the Geneious software v.9.1.8.

To create the reference *floR* sequence (*E. coli* AF231986.2), three different *floR* fragments were amplified using specific primers (Table S4) and the purified C4F *floR* amplicon as a template to introduce point-substitutions in the C4F *floR* gene to match the *floR* reference sequence. Reactions were carried out as before with Phire Hot Start II PCR Master Mix but using 70 °C as annealing temperature and different extension times for each fragment (6.8 s for fragment 1; 4.6 s for fragment 2; 8.3 s for fragment 3). Fragment 1 and fragment 3 contained overhangs that overlapped with the pETDuet-1 sequence at MCS-1. PCR products were purified before ligation with In-Fusion seamless cloning (Takara) according to the manufacturer’s instructions. Clones were checked, and vectors were purified, sequenced and analyzed as described above. Positive clones with sequenced vectors were routinely maintained in LB plates with 100 µg/mL ampicillin and stored as 10% glycerol stocks at −80 °C.

### 3.8. Heterologous Expression of floR and Antimicrobial Susceptibility Determination

Selected vectors carrying analyzed *floR* sequences (variants) were sub-cloned into *E. coli* BL21 (DE3) chemically competent cells. Three positive clones for each vector were selected and used for subsequent analysis. Antimicrobial minimal inhibitory concentration (MIC) assays for *E. coli* strains and clones were determined using the broth microdilution method [70]. *E. coli* ATCC 25922, *E. coli* BL21 (DE3) wild-type and *E. coli* BL21 (DE3) with the empty pETDuet-1 vector were used as controls in MIC determinations. *E. coli* cells were grown overnight at 37 °C in LB broth supplemented with ampicillin (100 µg/mL) and IPTG (100 µM), if appropriate. Overnight cultures were adjusted to 0.5 McFarland in Mueller–Hinton broth (MHB) and used as an inoculum for the MIC assay, which was carried out in MHB supplemented with 100 µM IPTG and serial two-fold dilutions of the desired phenicol antibiotic. Growth was evaluated after 24 h of static incubation at 37 °C. At least five independent replicates with two to three technical replicates were conducted per *E. coli* strain.

## 4. Conclusions

Bacterial communities in seawater from salmon farms exposed to sublethal concentrations of FFC encode several phenicol resistance mechanisms. The most represented was the antibiotic efflux mechanism consisting of multidrug- and phenicol-specific ARGs. Consistent with the ubiquitous nature of the *floR* gene, the most abundant phenicol-specific antibiotic resistance gene found in both the C4F and C9F salmon farms was *floR*. This gene was conserved in the sequence and genetic context. Analysis of the flanking regions of *floR* suggested that the occurrence of the *floR* gene was most likely derived from Gammaproteobacteria, regulated by a LysR-type transcriptional regulator and mobilized by transposases. Although conserved, the sequences of the *floR* gene and protein had variants that were conserved at both sites and with site-specific variants. The sequence diverged by three to six amino acids between local variants (clones) and the reference FloR sequence from *E. coli*, and these aminoacidic changes translated into both increases and decreases in phenotypic resistance to phenicol antibiotics. In general, changes in conserved motifs and transmembrane domains (motif C, TMS7, TMS12) and substitution of non-polar amino acids with hydrophobic side chains for polar or large-side chain amino acids generated a considerable decrease in antibiotic resistance. Thus, the sequence variations displayed phenotypic differences in antibiotic resistance. The coexistence of high- and low-resistance variants of *floR* in the environmental bacteria suggests that the salmon farming environment allows for the accumulation of point mutations that are maintained in bacterial populations.

## Figures and Tables

**Table 1:** Sampling location, fish farm characteristics and physicochemical properties of the surface seawater at the time of sampling.

Site Characteristics	C4F	C9F
Sample type	marine bacteria, seawater	marine bacteria, seawater
Date of sample collection	13 May 2022	20 October 2022
Farm depth (m)	16	20
Fish species	*Salmo salar*	*Oncorhynchus kisutch*
Time in sea water	7 months	6 months
Fish avg. weight	2.978 kg	2.081 kg
Fish density	6.90 kg/m^3^	5.4 kg/m^3^
SRS diagnosis (at sampling time)	yes	no
Treatments (last 3 months)	florfenicol (oral)	florfenicol (oral)
Temp. [°C]	10.87	11.59
pH	7.79	7.33
Conductivity EC [µS/cm]	43,910	4797
Total dissolved solids [ppm]	21,960	2398
Salinity [psu]	28.29	2.59
Oxygen D.O. [%]	97.9	99.6

**Table 2:** Identification of contigs containing the *floR* sequence by the RGI tool and sequence alignment to the identified *floR* variants (variant search).

Sample	Contig Name	RGI Percent Identity	RGI Coverage	Variant Search Percent Identity
C4F	NODE_20705	99.26	100	100
	NODE_606248	90.91	29.7	not found
	NODE_566496	not found	not found	88.7
	NODE_1421360	not found	not found	85.6
C9F	NODE_34943	99	100	100
	NODE_475405	72.73	20.5	not found
	NODE_702926	72.73	6.68	not found
	NODE_1375194	71.43	21.78	not found
	NODE_1375194	71.43	21.78	not found

## Data Availability

Metagenomic data were uploaded to the repository (National Center for Biotechnology Information, NCBI) that can be found at http://www.ncbi.nlm.nih.gov/bioproject/1197554 (accession no. PRJNA1197554).

## Supplementary Materials

The following supporting information can be downloaded at: https://www.mdpi.com/article/10.3390/antibiotics14020122/s1, Figure S1: Antibiotic susceptibility tests (MIC results); Table S1: RGI results; Table S2: *floR* sequences; Table S3: mOTUs; Table S4: Primers used in this study.
