# Supplementary material for: From Metagenomes to Functional Expression of Resistance: floR Gene Diversity in Bacteria from Salmon Farms
Source: Antibiotics (Basel). 2025 Jan 24;14(2):122. doi: 10.3390/antibiotics14020122 (PMC11851438; doi:10.3390/antibiotics14020122)
Supplement: Supplementary file 1 [file antibiotics-14-00122-s001.zip › Supplementary material/FigureS1.MICs.pdf]

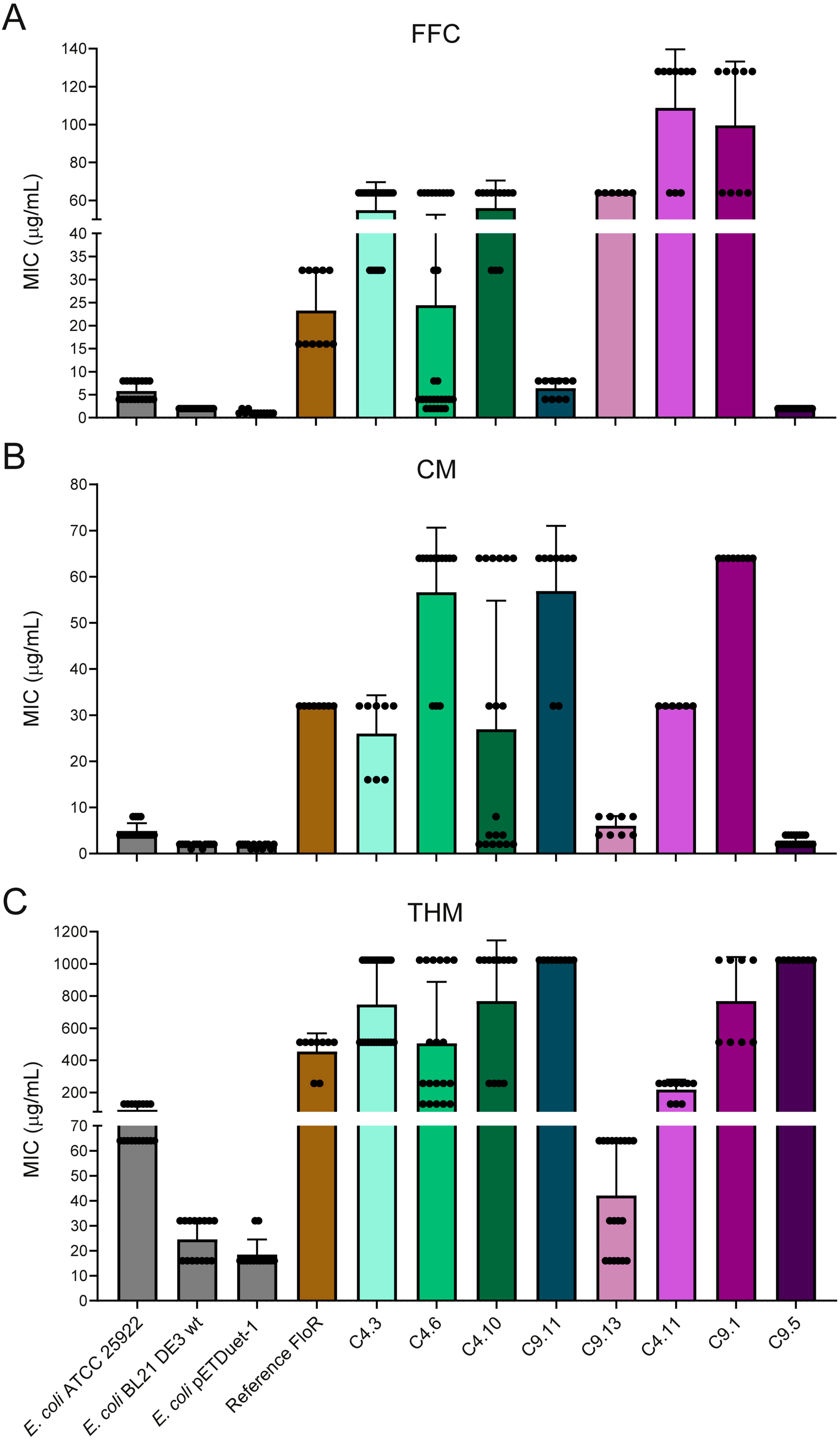

Figure S1. Antibiotic susceptibility test of the *E. coli* strains with or without cloned *floR* gene to phenicol antibiotics. MIC results for florfenicol (FFC) antibiotic (A, n= 5-14 independent replicates per strain), for chloramphenicol (CM) antibiotic (B, n= 4-10 independent replicates per strain), and for thiamphenicol (THM) antibiotic (C, n= 4-13 independent replicates per strain). *E. coli* ATCC 25922, *E. coli* BL21 (DE3) wild-type (wt), and *E. coli* BL21 (DE3) with the empty pETDuet-1 vector were used as controls. *E. coli* BL21 (DE3) was used as host for cloned vectors. Reference FloR corresponds to *E. coli* AF231986.2 protein sequence.
